# Counter-Irritation

**Published:** 1871-02

**Authors:** J. E. Hendricks

**Affiliations:** DesMoines, Iowa


### COUNTER-IRRITATION.

BY J. E. HENDRICKS, M.D., of DesMoines, Iowa.

Some author, whom I recollect having read, undertakes to *ridicule* the principle of counter-irritation by comparing the practice to that of a man, who, when his front door is assailed by robbers, commences with an axe to hew down the back door. It is obvious, from the illustration used, that this author has no rational conception of the *nature* of disease, nor of the *action* of its appropriate remedy.

Since it is a demonstrated fact that the molecules of all forms of matter have a rapid motion among themselves, and since, from the laws of heat, we infer that different substances have different molecular motions, it is entirely consistent to conclude that the different organs and tissues of the animal organization have each a molecular motion peculiar to itself, and which differs in some respects from that of all other organs and tissues. This motion I will call the *normal* molecular motion of the tissue.

It is apparent that during the life of every animal organization, the normal motions of the tissues of which it is composed are subject to various sources of disturbance from without. These disturbing causes, in many instances, so change the normal molecular motion of the tissues that the *harmony* of motion in contiguous tissues, which is the essential characteristic of the state of *health*, is destroyed, and the resulting condition of the organism we call the state of *disease.*

This hypothesis of the nature of disease, though not an ascertained fact, harmonizes entirely with what we know of the conditions of health and disease; and, moreover, it accords with all the recent developments of physical science; and presents something physical and tangible to think and talk about, while searching for a remedy for the cure of disease.

Granting our hypothesis, and assuming, for instance, that in *iritis* the normal molecular motions of the iris have been so changed by the disturbing causes that, in the diseased state of the tissue, the molecular motion is more rapid than in its healthy state—that is, the disease is the result of an *increase* of the normal molecular motion of the iris; the *indication* for the cure of *iritis* would, therefore, obviously be to diminish the molecular motion of the diseased tissue. But material motion can only be arrested by material contact, or *interference.*

The only rational treatment will, therefore, be to administer some substance whose molecules have a less rapid motion than those of the diseased tissue, and which will come in contact with the molecules of the tissue, and, by *interference*, diminish their velocity. Let it be granted that the molecules of sub. muriate of mercury have such a motion. Then, because if the medicine is introduced into the stomach, it will enter the circulation and be brought in contact with every tissue in the organization, it will, therefore, *necessarily* come in contact with the molecules constituting the iris, and, by interference, diminish their velocity. But calomel is found to be a specific for *iritis;* hence, if our hypothesis is true, our assumptions are correct, and we have in the administration of the medicine, a *rational* treatment of the disease. But, in many cases, we do not *know* a medicine whose molecules by interference will tend to correct the motions of those of the diseased tissue. What is the indication for a cure in such cases? Obviously, *counter-irritation.* That is, if we cannot act directly on the molecules of the diseased tissue, we may so change the molecular motion of a contiguous tissue that they, by interference with the molecules of the diseased tissue, will induce in them a change *toward* their normal rate; and thus we do, indirectly, precisely the same thing which we accomplished directly in the first case.

